# Development of a Health Information Technology Tool for Behavior Change to Address Obesity and Prevent Chronic Disease Among Adolescents: Designing for Dissemination and Sustainment Using the ORBIT Model

**DOI:** 10.3389/fdgth.2021.648777

**Published:** 2021-03-10

**Authors:** Maura M. Kepper, Callie Walsh-Bailey, Ross C. Brownson, Bethany M. Kwan, Elaine H. Morrato, Jane Garbutt, Lisa de las Fuentes, Russell E. Glasgow, Marcelo A. Lopetegui, Randi Foraker

**Affiliations:** ^1^Prevention Research Center, Brown School, Washington University in St. Louis, St. Louis, MO, United States; ^2^Institute for Public Health, Washington University in St. Louis, St. Louis, MO, United States; ^3^Division of Public Health Sciences, Department of Surgery, Alvin J. Siteman Cancer Center, Washington University School of Medicine, Washington University in St. Louis, St. Louis, MO, United States; ^4^Department of Family Medicine, Adult & Child Consortium for Health Outcomes Research & Delivery Science, University of Colorado Anschutz Medical Camps, Aurora, CO, United States; ^5^Parkinson School of Health Sciences and Public Health, Loyola University Chicago, Maywood, IL, United States; ^6^Division of General Medical Sciences, Department of Medicine, Washington University School of Medicine, St. Louis, MO, United States; ^7^Cardiovascular Division, Department of Medicine, Washington University School of Medicine, St. Louis, MO, United States; ^8^Centro de Informática Biomédica, Instituto de Ciencias e Innovación en Medicina (ICIM), Facultad de Medicina Clínica Alemana, Universidad del Desarrollo, Santiago, Chile; ^9^Center for Population Health Informatics, Institute for Informatics, Washington University in St. Louis, St. Louis, MO, United States

**Keywords:** health information technology, obesity, behavior change, sustainability, stakeholder engagement

## Abstract

Health information technology (HIT) has not been broadly adopted for use in outpatient healthcare settings to effectively address obesity in youth, especially among disadvantaged populations that face greater barriers to good health. A well-designed HIT tool can deliver behavior change recommendations and provide community resources to address this gap, and the Obesity-Related Behavioral Intervention Trials (ORBIT) model can guide its development and refinement. This article reports the application of the ORBIT model to (1) describe the characteristics and design of a novel HIT tool (the PREVENT tool) using behavioral theory, (2) illustrate the use of stakeholder-centered “designing for dissemination and sustainability” principles, and (3) discuss the practical implications and directions for future research. Two types of stakeholder engagement (customer discovery and user testing) were conducted with end users (outpatient healthcare teams). Customer discovery interviews (*n* = 20) informed PREVENT tool components and intervention targets by identifying (1) what healthcare teams (e.g., physicians, dietitians) identified as their most important “jobs to be done” in helping adolescents who are overweight/obese adopt healthy behaviors, (2) their most critical “pains” and “gains” related to overweight/obesity treatment, and (3) how they define success compared to competing alternatives. Interviews revealed the need for a tool to help healthcare teams efficiently deliver tailored, evidence-based behavior change recommendations, motivate patients, and follow-up with patients within the constraints of clinic schedules and workflows. The PREVENT tool was developed to meet these needs. It facilitates prevention discussions, delivers tailored, evidence-based recommendations for physical activity and food intake, includes an interactive map of community resources to support behavior change, and automates patient follow-up. Based on Self-Determination Theory, the PREVENT tool engages the patient to encourage competence and autonomy to motivate behavior change. The use of this intentional, user-centered design process should increase the likelihood of the intended outcomes (e.g., behavior change, weight stabilization/loss) and ultimately increase uptake, implementation success, and long-term results. After initial tool development, user-testing interviews (*n* = 13) were conducted using a think-aloud protocol that provided insight into users' (i.e., healthcare teams) cognitive processes, attitudes, and challenges when using the tool. Overall, the PREVENT tool was perceived to be useful, well-organized, and visually appealing.

## Introduction

Obesity among youth in the United States, particularly those of low-socioeconomic status (SES), is a major public health concern that puts youth at risk for poor cardiovascular health (CVH) (1–3). There is increasing evidence that intervening on and maintaining ideal CVH factors (e.g., weight, physical activity, and healthy food intake) during adolescence delays the progression toward clinical disease and will yield considerable health benefits in adulthood (4). Yet, obesity remains underdocumented and undertreated in this age group, and there is a paucity of effective, scalable, and sustainable solutions (5). Less than half of children who see a pediatrician receive body mass index (BMI) screening and/or counseling for diet and physical activity as recommended by the American Academy of Pediatrics (6–9). Referral to dieticians, behavioral counseling, or other community resources [e.g., Women, Infant and Children (WIC) clinics, farmer's markets, fitness centers] occurs among a minority of adolescents who are overweight/obese; referrals, when completed, support positive changes in healthy eating and physical activity (10–14). Marginalized populations who face the greatest barriers and social determinants of health (SDOH)-related needs especially benefit from resource referral and are more severely impacted by these missed opportunities (15). The inclusion of tailored community resources allows the behavioral intervention to be adapted to an individual's environment and needs, and has resulted in improvements in BMI when delivered at the point of care (14, 16, 17).

Health information technology (HIT) has the potential to improve the quality, efficiency, consistency, and availability of healthcare across diverse populations (8, 18–20). Despite rapid emergence, few HIT tools have been designed to support the delivery of behavior change recommendations and community resources at the point of care, particularly among children and adolescents (18, 20). Furthermore, the production of such technology does not guarantee implementation, let alone sustained use. Several factors may impact the sustainability of HIT: (1) end-user adoption and utilization, (2) interface and usability issues that may negatively impact workflow and productivity, and (3) restrictions on vendor behaviors that make it challenging to leverage and integrate multiple data sources and types (19, 21, 22). To overcome these challenges, HIT should be feasible and efficient and account for the needs, contexts, workflows, and resources of the end users and decision makers.

Designing for Dissemination, Implementation, and Sustainability (D4DIS) refers to developing interventions that are closely aligned with the needs of end users and the intended context for use (23–25). The use of D4DIS strategies in conceptualization and development of health interventions and technologies improves fit with adopters and contexts to promote later success and sustainability (24). Increased participation of stakeholders (e.g., healthcare teams) in the development of interventions is a core principle of D4DIS. Combined with stakeholder engagement, the use of dissemination and implementation (D&I) science frameworks can enhance adoption, reach, implementation, and sustainment of interventions (26–29). The Reach, Effectiveness, Adoption, Implementation, Maintenance (RE-AIM) framework has been widely used in the planning, design, and evaluation of behavior change interventions (30–33). RE-AIM was developed to ensure that research findings were more generalizable by balancing internal and external validity when developing and testing interventions (34).

D4DIS strategies can inform intervention design features (e.g., the significant clinical question, what the intervention will entail, meaningful outcomes), implementation and sustainment strategies, and dissemination channels. We used D4DIS strategies (stakeholder engagement and the RE-AIM framework) within the process of the Obesity-Related Behavioral Intervention Trials (ORBIT) model (35) to develop a novel behavior change HIT (the PREVENT tool) intervention for pediatric weight management. The intent was to use D4DIS strategies within the ORBIT Model to develop an effective behavioral intervention that fits within its intended clinical context to speed translation into practice (35). The ORBIT model provides the behavioral research field a well-defined process for developing a new intervention or tool that emphasizes development and refinement in the early phase to strengthen behavioral treatments, encourage their testing in rigorous efficacy and effectiveness trials, promote success, and foster dissemination in clinical practice. The ORBIT model has been used in the development of several interventions targeting physical activity, nutrition, and/or healthy weight (36–39).

A major component of the ORBIT model is underpinning interventions with basic and social sciences research, which includes the use of behavioral theory, to increase effectiveness and allow developers to target theoretically derived drivers of behavior (35, 40). Self-Determination Theory (SDT) is a widely applied behavioral theory concerned with how the psychological needs of autonomy, relatedness, competence, and one's social environment can support or undermine an individual's motivation to perform a behavior. SDT identifies 4 different types of motivation categorized as autonomous (intrinsic and identified regulation) and controlled (external and introjected regulation). Intrinsic motivation refers to performing a behavior based on the inherent satisfaction or enjoyment of a behavior. Additionally, a person may be motivated by the behavior's personal value and utility (e.g., being active because one values the health benefits). Autonomous motivators have been shown to be more influential than controlled motivators for the promotion and sustainment of physical activity and healthy eating among children and adolescents (41–45). SDT principles (e.g., supporting participant choice, emphasizing behavior change that is enjoyable and feasible) shown to make interventions more successful at encouraging behavior change were applied in the development of PREVENT (46). Psychological needs support as described by SDT aids in promoting health behavior change in adolescents (47). Furthermore, the SDT informs recommendations for how to administer the PREVENT intervention with an autonomy-supportive climate of mutual understanding, trust, and shared decision making between the patient and healthcare team member, which increases patient satisfaction and treatment adherence (48–50).

The purposes of this report are to apply the ORBIT model to (1) describe the characteristics and design of the PREVENT tool using behavioral theory, (2) illustrate the use of D4DIS principles within the design Phase I of the ORBIT Model, and (3) discuss the practical implications and directions for future research (ORBIT Phase II–IV). This article will present two types of stakeholder engagement (customer discovery and user testing) used to design the PREVENT tool.

## Methods

**Figure 1:**
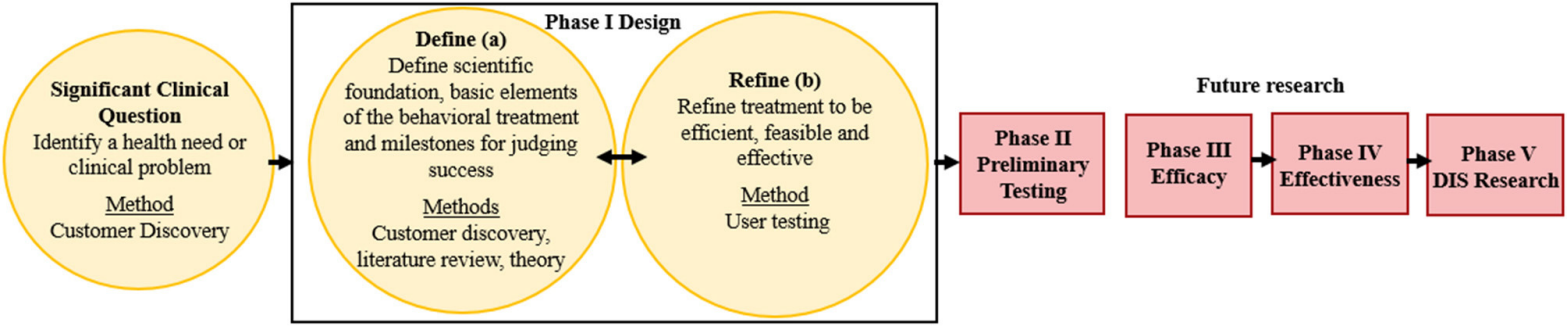
Application of the ORBIT Model for PREVENT Tool Development. Yellow boxes indicate completed research; red boxes are future research; DIS: dissemination, implementation, and sustainability. Figure adapted from Czajkowski et al. (35).

The ORBIT model guided the development of a behavior change HIT tool (the PREVENT tool) designed to help pediatric healthcare teams (e.g., physicians, dietitians) address overweight and obesity within routine care. This model proposes guidelines for developing behavioral treatments from the inception of an innovative approach through efficacy testing (Figure 1). Our goal is to scale and sustain the PREVENT tool across several clinical settings and patient groups; therefore, we engaged a diverse set of healthcare team members, and our model (Figure 1) adds an additional fifth phase of dissemination, implementation, and sustainability (DIS) research. The ORBIT model informed key questions at each stage of development, our selection of methodologies, and milestones for pre-efficacy development and testing of our tool. This report presents the following stages of development: (1) identification of a significant clinical question, (2) design of the behavioral treatment using behavioral theory and intentional selection of treatment targets, and (3) refinement of the HIT tool to promote efficiency and acceptability to the target user (e.g., healthcare teams). To progress through these stages, we reviewed the literature and applied relevant theories and frameworks. In addition, we utilized qualitative methods for stakeholder engagement (customer discovery and user testing) that are necessary to develop interventions that meet the needs of adopters and overcome low uptake and maintenance of HITs. Institutional Review Board (IRB) approval (formal informed consent not required) was granted to perform customer discovery and user-testing interviews with healthcare team members (e.g., physicians, nurses, dieticians, behavioral counselors) recruited using snowball sampling methods within academic and community-based practice settings. Subsequent stages of development and testing will engage patients and families' to ensure that the tool meets their needs. Methods were selected from suggestions in the ORBIT model (35) and those used in D4DIS to facilitate the development of an effective, pragmatic, and sustainable solution to behavior change at the point of care.

### ORBIT Model: Identification of a Significant Clinical Question

The first step in the ORBIT model is to identify and articulate a health need or clinical problem requiring a solution and generate a specific hypothesis. The goals of explicitly identifying the clinical problem are to (1) include primary behavioral or clinical endpoints in Phase II Preliminary Testing, (2) prepare the Phase III Efficacy trial to test the benefit of the treatment on a clinically meaningful outcome, and (3) commit to achieving a sufficient, meaningful change (35). The ORBIT model does not advocate explicitly for the inclusion of implementation outcomes in preliminary testing. Therefore, we drew upon D4DIS to identify relevant implementation outcomes, using the RE-AIM framework to align with our goal of developing a pragmatic, sustainable intervention.

#### Customer Discovery

Customer discovery and value proposition design, a form of stakeholder engagement based on marketing and LeanStartup business methods (51), was used to understand the clinical problem and articulate the product's hypothesized unique value proposition relative to alternative options available to end user. Customer discovery has been applied by academic entrepreneurs and health researchers in sustaining health informatics innovation (27, 52). In a randomized trial, entrepreneurs who embraced the scientific process and tested value propositions through customer discovery increased the likelihood for sustainability (53). The National Institute of Standards and Technology endorses these methods to promote technology transfer at universities (54).

We used value proposition design methods described by Osterwalder et al. (55). Value proposition design is a technique used to ensure “problem-solution” fit in product development. A product (e.g., HIT tool) can derive value by helping the user: (1) achieve outcomes important to them and their job (“gain creators”) and (2) avoid poor outcomes, risks, and obstacles (“pain relievers”) (27). Customer discovery interviews (mean duration = 19 min) were conducted with 20 healthcare team members (e.g., physicians, nurses, dieticians, behavioral counselors) who represented the target users within primary care (*n* = 12) and academic specialty clinics (*n* = 8). Both primary and specialty pediatric clinics were selected to provide a diverse sample of clinical settings in order to develop a tool that may benefit a variety healthcare teams and reach diverse adolescent populations, including those of low SES. Through customer discovery, our research team identified (1) what healthcare teams' felt were their most important “jobs to be done” in helping adolescents with overweight/obesity adopt healthy behaviors, (2) critical “pains” and “gains” related to overweight/obesity treatment, and (3) how they would measure success. The goals of these interviews were to identify a need or clinical problem requiring a solution, articulate a strong value proposition, and identify outcome measures that are meaningful to healthcare teams to demonstrate success in their treatment of overweight/obesity among adolescent patients. A value proposition statement articulates how a product helps create desired gains and relieve pains for unsatisfied jobs to be done for a given customer segment. Product features that address such needs are more likely to be adopted (56).

### ORBIT Phase 1: Design

The goal of Phase I was to design the essential features of a behavioral treatment. Phase I is divided into two phases: Ia (Define) defines the scientific foundation and basic elements of the behavioral treatment and milestones for judging success, and Ib (Refine) refines the treatment to optimize efficiency and feasibility while promoting the intended clinical change. The ORBIT model advocates for the explicit identification of behavioral drivers that translate into intervention targets, an identification of methods by which these targets can be altered, and the translation of targets into quantifiable measures to evaluate the intervention's success (35). Intervention components and milestones were identified in customer discovery interviews and further refined from the literature. SDT was applied to base the identification of treatment targets (e.g., motivation) in behavioral theory, align the PREVENT tool to these targets, and identify process measures that assess the impact on patient motivation. User testing was used in ORBIT Phase Ib to ensure that the PREVENT tool is efficient, easy to use, and fit for purpose among end users (healthcare teams).

#### User Testing

User testing evolved in the disciplines of human–computer interaction and system design, thus focuses on the extent to which users find the tool easy to use and effective for the tasks they need to perform (57). A think-aloud protocol (58) was employed to conduct user testing interviews with 13 healthcare team members (8 physicians, 2 nurses, 2 dieticians, and 1 behavioral specialist) who represented target users. This protocol guided participants to verbalize their thoughts while engaging with the PREVENT tool, which provided insight into users' cognitive processes and attitudes toward the tool (58). If the participant stopped talking, the interviewer probed with open-ended questions (e.g., “what are your thoughts right now?”). The participant accessed tool using a web platform and shared their screen with the interviewer. All interviews were recorded including both audio and visual displays to allow coders to identify problems expressed verbally and through participants' interaction with the tool. At the end of each interview, the Zoom polling function was used to collect responses to seven team-developed items that assessed healthcare team members' perceived usability and usefulness of the PREVENT tool on a 5-point Likert scale (1 = strongly disagree to 5 = strongly agree).

### Qualitative Data Analysis

Customer discovery and user-testing interviews were analyzed separately using the same techniques. Rapid qualitative analysis methods (59) were applied by 2 raters (MMK and CWB) using the following steps: (1) create a matrix, (2) establish interrater reliability by independently coding 3 interviews and generating consensus, (3) independently code remaining interviews, and (4) summarize themes. The use of customer discovery results were guided by Strategyzer learning cards that guide the translation of observations into learnings and decisions/actions for development (55, 60). Insights informed the value proposition statement, priority features of a HIT tool, and selection of meaningful outcome measures.

## Results

### Identification of the Significant Clinical Question

Customer discovery insights and supportive provider quotes are summarized in Table 1 by interview topic: provider's roles/jobs to be done, their pains and gains in those jobs, and measures of success. Healthcare team members identified that treating comorbidities, delivering lifestyle (e.g., physical activity, nutrition, sleep) counseling, and educating their patients and families was a major part of their role in helping adolescents with overweight/obesity. Their goal was to emphasize the importance of lifestyle intervention; discuss future risks or benefits of their health status; help patients set achievable, tailored goals; and provide resources to help patients meet their goals and follow-up with patients. Healthcare team members expressed difficulty in addressing all concerns within the clinic visit timeframe and following up with patients on a regular basis. Patients often live long distances from the clinic or lack transportation, making frequent in-person follow-up challenging. Healthcare team members lack educational resources, care team personnel (e.g., dieticians), and evidence-based behavior change programs. Difficulty measuring health behaviors limited provider ability to tailor recommendations and often resulted in healthcare teams spending substantial time to understand patients' current behaviors and surrounding circumstances (e.g., home and neighborhood environment). Additionally, lack of data limits healthcare teams' and patients' ability to appreciate if progress is (or is not) being made. Healthcare teams are challenged by the sensitivity and stigma of this topic and lack of patients' and parents' motivation, readiness to change, and self-efficacy (i.e., confidence) to change behaviors. Healthcare team members acknowledged the impact of their patients' external context (e.g., food and physical activity environment, socioeconomic factors) and that they were not equipped/knowledgeable of resources to address these issues. Change in behaviors (e.g., goal progress, increases in physical activity) and weight loss or stabilization were key indicators of success. To a lesser extent, healthcare team members were interested in (1) whether goals are set during the care visit, (2) diagnosis/treatment of comorbidities (reported primarily by specialty clinic teams), (3) patient knowledge (e.g., understanding of growth charts, importance of lifestyle factors), and (4) completion of follow-up visits.

**Table 1:** Summary of customer discovery results.

	**Main themes**	**Quotes**
Jobs	• Provide lifestyle coaching (physical activity, healthy eating, and sleep); help patient's set goals • Understand current behaviors and potential barriers to change (e.g., lack of motivation, family dynamics) • Educate patients (e.g., future health risks, importance of physical activity and nutrition, healthy foods) • Treat comorbidities • Recommend community resources or comprehensive obesity care clinics • Maintain relationships	“I try not to focus too much on the number on the scale but more so just the habits they should adopt to be healthy in general whether it is through healthy eating, limiting screen-time, encouraging more physical activity and also trying to address any mental triggers for unhealthy eating or poor lifestyle habits.” “I empower them to want to have a healthy lifestyle, and talk to them about how your health is for the rest of your life.”
Pains/Gains	• Lack of time within visit and for follow-up • Difficulty assessing health behaviors and family dynamics • Inability to see/measure progress • Patients and families lack motivation • Lack of provider knowledge of available resources accessible to their patients • Patient's environment/social determinants of health	“A lot of times they aren't coming in for follow ups, which is frustrating because I can't help them if they aren't coming to see me.” “I really enjoy working with these patients but it does get frustrating that it is hard to see that you are making a difference.” “On day to day basis, I feel like we don't see the changes I would hope to see as a healthcare provider…We don't see that our hard work is working or the fruits of labor.” “The…issue, I think is lack of motivation because most often when we see an obese adolescent their caretaker…is also obese…they're not viewed as being abnormal, they're not viewed as being at risk.” “I may or may not know where they live but I certainly don't know what resources are available to them.” “If the EHR automatically gave me resources for the patient…. It would potentially allow me to more quickly give that intervention.”
Metrics of success	• Change in behaviors (e.g., goal progress) • Weight loss or stabilization • Delivery of goals/counseling • Diagnosis/treatment of comorbidities • Patient knowledge • Completion of follow-up visits	“I try to have them understand that lifestyle intervention is the number one treatment…the first thing I like to achieve is you have to change your way of thinking and we have to change some behaviors here to accomplish what we're trying to accomplish.” “Anything from 3 to 5% of their total body weight percentage lost would be a great goal for them to achieve, 5–10% would be ideal.” “Seeing their heart rate not jump up quite as much, seeing them be a little more physically fit are a good way to track some of those physical activities, but most of it is patient report…. At the end of all of my sessions with patients I try to set goals with them…trying to check in and see how they're doing with their goals at every visit.”

In summary, there was clinical need for a tool to help healthcare teams efficiently deliver tailored, evidence-based behavior change recommendations and follow-up with patients. The majority of healthcare team members were not currently using nor had previously used any HIT tool that met these needs. To promote meaningful change, the tool should address the patient's motivation to change their health behaviors and overcome barriers (e.g., lack of community resources) to healthy behaviors. The generation and use of data are necessary to measure and communicate progress to the patient and the provider and inform care decisions. The initial value proposition was “our PREVENT tool delivers tailored, evidence-based physical activity and nutrition prescriptions that account for SDOH to help you improve your care beyond generic recommendations within the time constraints of a patient encounter.” Based on the customer discovery, the value proposition was modified to: “the PREVENT tool helps healthcare teams pragmatically address obesity among adolescents by displaying pertinent health data; delivering personalized, evidence-based lifestyle change recommendations; providing community resources; and automating virtual patient follow-up.”

### PREVENT Tool Design (Phase I)

#### Characteristics of the PREVENT Tool (Phase Ia)

The PREVENT tool was designed for healthcare team members to use on a tablet or computer during clinical care visits with adolescent (12–19 years of age) patients who are overweight or obese. The tool addresses a clinical need by helping healthcare teams with their job to discuss health and prevention (Step 1); deliver tailored, evidence-based recommendations for physical activity and healthy food intake (Step 2); recommend community resources to support behavior change (Step 3); and provide detailed recommendations and education and maintain relationships with patients electronically (Step 4). Motivating patients was identified by healthcare team members as a critical treatment target to successfully promote physical activity and healthy eating within the short patient–provider encounter. Features of the PREVENT tool were designed following SDT Theory basic tenets to help healthcare team members motivate patients by improving their competence and autonomy through choice and self-initiation. All healthcare team members will be trained prior to using the PREVENT tool on the use of neutral language during patient–provider interactions (e.g., “may” and “could” not “should” or “must') to further support autonomy (61, 62).

**Figure 2:**
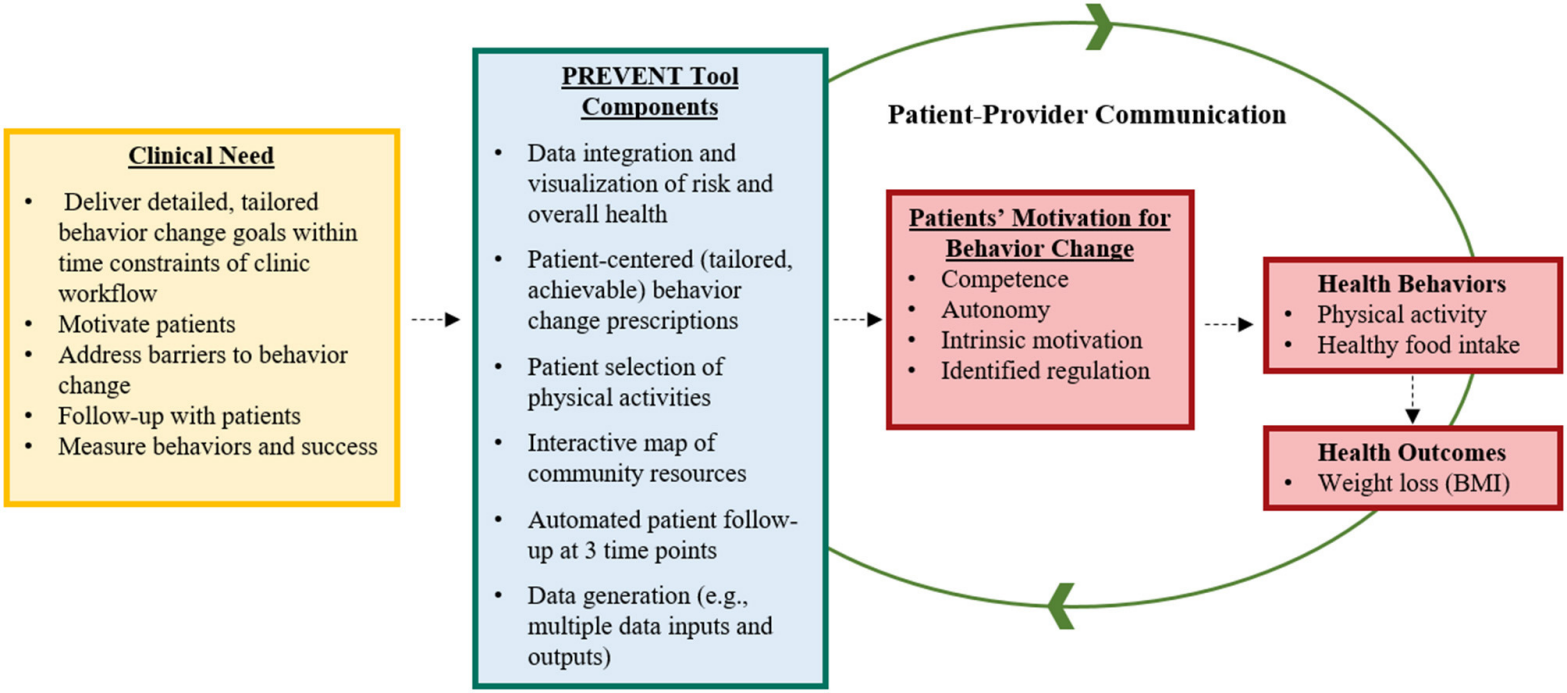
PREVENT tool pathway of development and hypothesized impact.

In addition to facilitating intervention, the tool generates data to inform care and monitor patient behavioral and clinical progress to facilitate ongoing communication between the patient and provider. In addition to an initial screening, patients will report whether they are meeting their physical activity and healthy food intake goals monthly. Survey responses are displayed immediately within the PREVENT tool to enable healthcare team members to view patient progress and potentially adjust patient care (e.g., schedule in-person clinic visit). Based on responses, patients will receive automated email messages with updated progressive goals and motivation. We hypothesize that the PREVENT tool will improve patient motivation to make behavior change, improve their physical activity and food intake behaviors, and stabilize weight gain or generate weight loss among adolescents (Figure 2).

**Figure 3:**
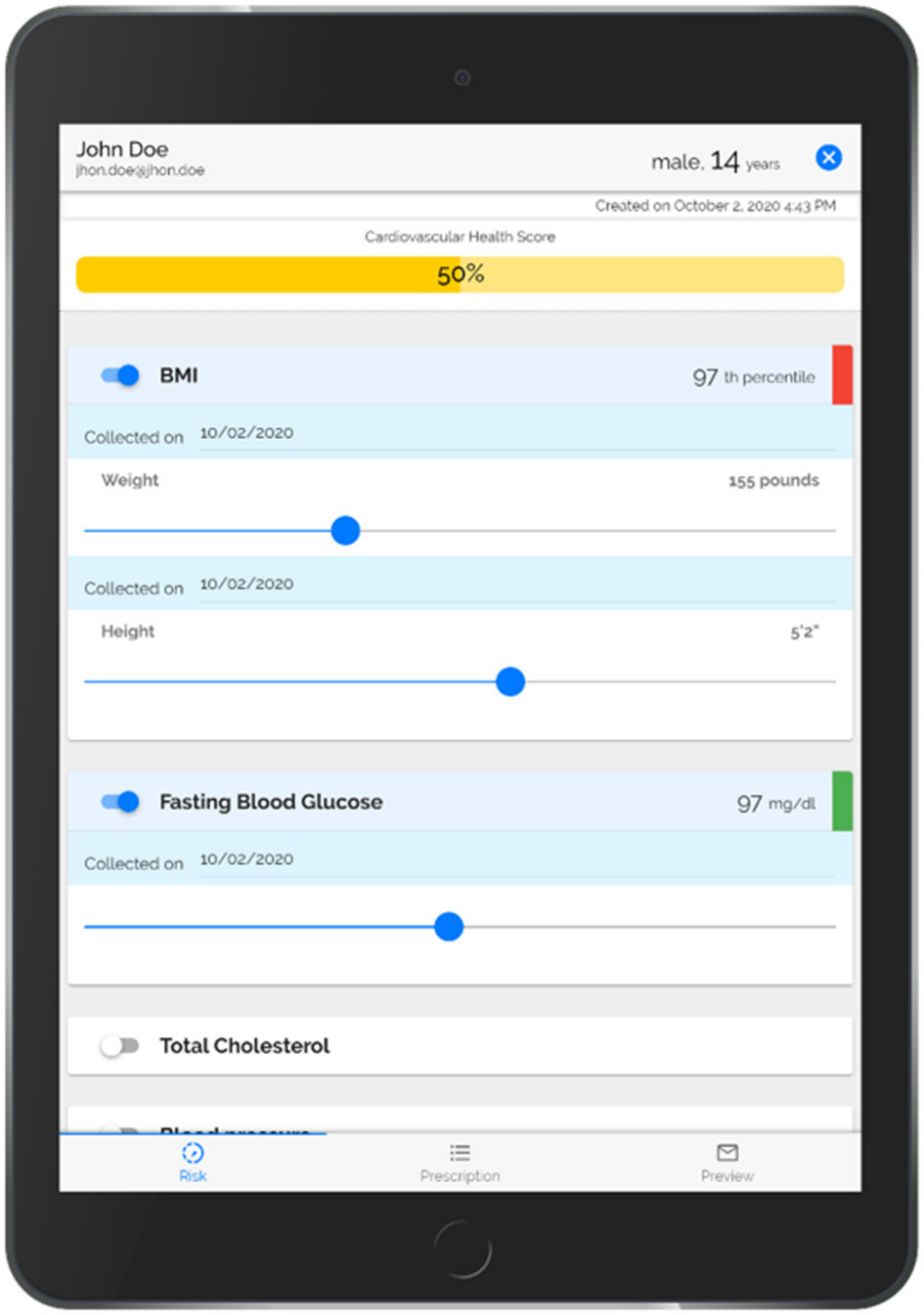
PREVENT tool step 1, discuss prevention and cardiovascular health.

**Step 1**. PREVENT uses a robust informatics approach to automatically populate and visually display American Heart Association (AHA)'s Life's Simple 7 Risk Factors (BMI percentile, blood pressure, cholesterol level, glucose level, smoking status, physical inactivity, and food intake) using patients' EHR and survey data (Figure 3). PREVENT integrates information from multiple input modalities and locations across the EHR. A previously described and validated scoring system (63–66) calculates an overall cardiovascular health score based on the AHA's Life Simple 7 definitions of ideal (green), intermediate (yellow) and poor (red) health. Child and adolescent cut points (67, 68) were applied to categorize and score each risk factor as follows: 2 points for each factor at ideal levels, 1 point for each factor classified as intermediate, and 0 points for each factor indicating poor health. The points are summed and divided by the total possible number of points (maximum of 14 points when all risk factors are used). Each risk factor is displayed with slider bars to allow the provider to show the patient the impact of small changes on their overall CVH score to facilitate prevention discussions. Moving the slider bar (e.g., increasing physical activity minutes) changes the color coding of the factor and recalculates the overall health score to emphasize the importance of health behaviors on overall CVH. Delivering a clear rationale for adopting the health behavior (e.g., impact on CVH) appeals to the SDT tenet of “identified regulation” to motivate patients. As adolescents may be more motivated by short-term impacts, the healthcare team member will be encouraged to discuss short-term benefits of behavior change (e.g., improved sleep, mental health) during the patient encounter. This approach has been tested in adults and facilitated more prevention discussions, was accepted by healthcare providers, and improved patients' BMI (65, 66). In addition to the Life Simple 7 Risk Factors, the tool collects and displays a patient's willingness to change their physical activity and food intake behaviors so that prevention discussions can target the behaviors most likely to improve.

**Step 2**. Tailored physical activity and food intake recommendations are automatically generated within the PREVENT tool based on the patient's risk profile (BMI percentile and health behaviors) using evidence-based behavioral recommendations [Trim Kids (69), the Stoplight Diet (70)] for youth. The recommendations are specific to the patient, ramp up gradually over time, and allow for patient preference to ensure they are realistic and achievable to promote patient competence. Healthcare team members and patients may decide to focus on a limited number of behaviors (e.g., focus on reducing unhealthy snacking) if this is deemed more realistic than targeting multiple behaviors. The tool follows up with patients to determine whether they are meeting their goals and delivers these data to the healthcare team to facilitate cyclical communication. The physical activity goals follow a moderate-intensity, progressively dosed (e.g., increasing activity by 10% each week) program that is tailored to the individual needs of youth with different levels of obesity (69). The recommended intensity, duration, and frequency are specific to the patient's obesity level (overweight, obese, or severely obese) and current physical activity status (inactive, somewhat active, moderately active, or active). For example, severely obese, inactive patients have shorter beginning durations and frequencies than do the overweight, inactive patients. These recommendations align with the United States Guidelines for Physical Activity, the American Academy of Pediatrics, and the American Heart Association (71–73). Additionally, the tool includes a menu of youth activities (e.g., walking the dog, swimming, playing basketball) to allow the patient to select activities they enjoy (use of intrinsic motivation) and feel competent to achieve their physical activity goals. These activities have been chosen because they are safe and effective for overweight or obese youth and promote light, moderate, or vigorous physical activity based on the Compendium for Physical Activity (74, 75). The Stoplight Diet was used as a framework for promoting dietary changes for 5 key eating behaviors: (1) fruit intake, (2) vegetable intake, (3) whole grains, (4) sugar sweetened beverage consumption, and (5) snacking behaviors (76). Recommendations are provided only for problem behaviors indicated in the patient survey (70). The Stoplight Diet was developed for children and adolescent populations and provides a simple, easily understood yet effective approach that may be feasible to communicate by a variety of healthcare team members and within the time constraints of clinical care visits (70). Patients are encouraged to increase their intake of low in fat, high nutrition density “green” foods and to decrease their intake of low in nutrient density “red” foods. The PREVENT tool delivers lists of green “anytime” foods, yellow “sometimes” foods, and red “rarely” foods for each target behavior (e.g., vegetable intake, snacking) to allow patients autonomy in their choice of healthy food options.

**Figure 4:**
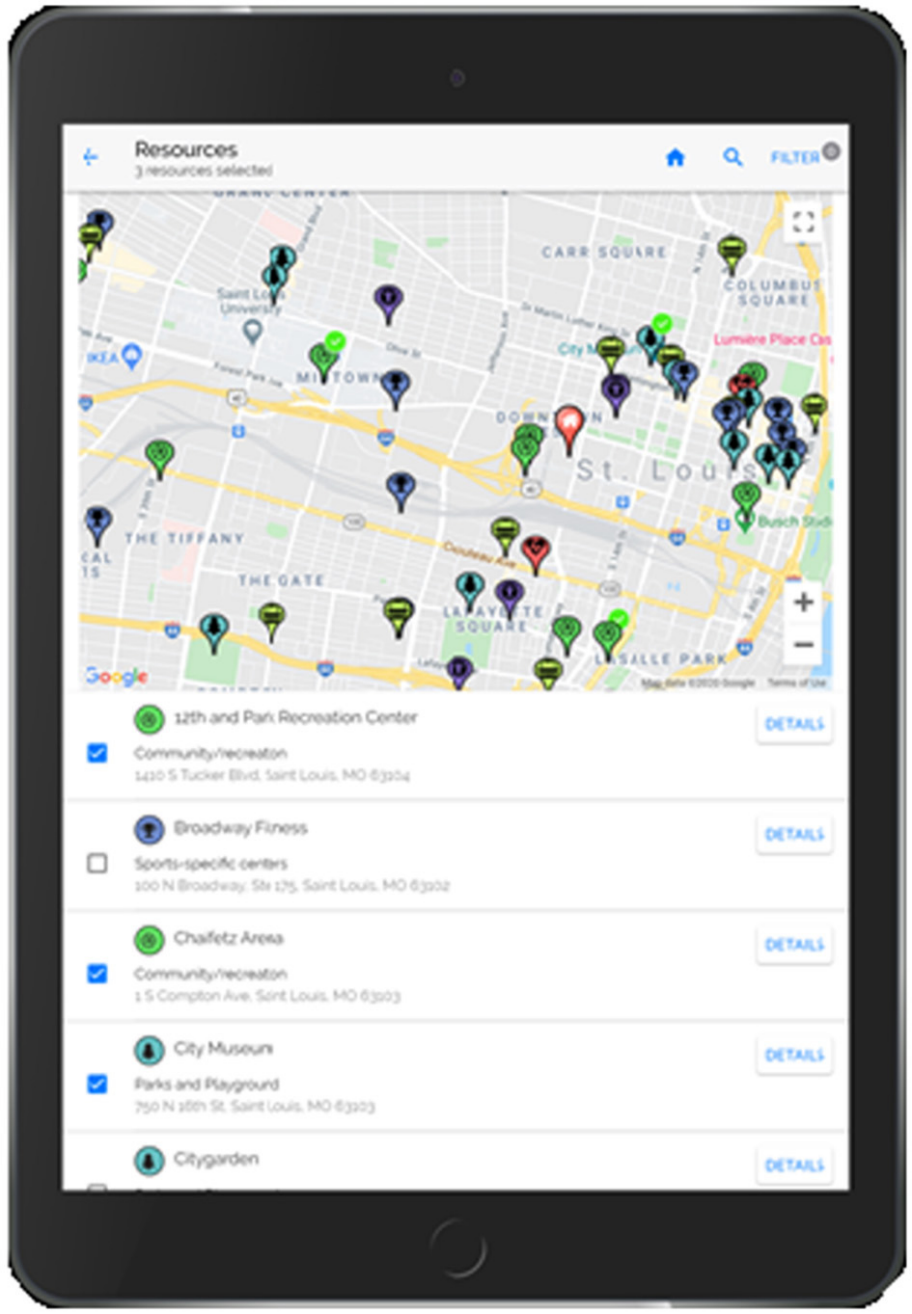
PREVENT tool step 3 community resource map.

**Step 3**. An interactive map of community resources (Figure 4) is included in the PREVENT tool to allow the patient to select resources to support the recommended behavior change. This component increases patient autonomy (choice/control) and capacity by including resources that are accessible (e.g., near their home, available via public transport) and perceived as helpful by the patient. The PREVENT tool connects to YELP via their application programming interfaces (API) and retrieves the following resources: weight management programs, parks and playgrounds, fitness/sports centers, community/recreation centers, grocery stores, farmer's markets, community-supported agricultures (CSAs), food pantries, and community gardens. YELP resources were validated and subsidized with resource lists generated manually from local organizations (e.g., parks and recreation departments, Missouri Coalition for the Environment) and internet searching. Initially, the map is zoomed to the patient's home address but can be moved or searched to display resources in other areas (e.g., near the patient's school). Directions can be generated via car, public transportation, or foot to allow the patient to determine if the resource is readily accessible. This map also provides detailed information on each resource, including hours of operation, contact information, and amenities (e.g., play equipment within the park).

**Step 4**. The provider sends a “prescription” to the patient and/or parent electronically via email and/or text message from the PREVENT tool. The patient identifies their preferred method of communication within the tool. This “prescription” includes the tailored behavior change goals, selected activities, community resources, and educational information (e.g., additional information on the Stoplight Diet, health benefits of physical activity and healthy food intake, tips for parents and families). The inclusion of educational material is important to increase patient's competence to reach behavior change recommendations. A link to the interactive community resource map is included to allow the patient to identify additional resources after they leave the clinic. All information is presented using appealing visualization and an appropriate literacy level to facilitate patient understanding. PREVENT increases provider contact with the patient and generates data on behavior change with three automatic email or SMS text message check-ins after the patient's clinic visit. Patients are asked to indicate whether they are meeting their physical activity and food intake goals using a 5-point Likert scale (never to always). Patients are provided encouragement and new goals based on their response so that goals are progressive and attainable.

#### Selected Outcomes and Measures for Subsequent Preliminary Testing (Phase 1a)

Measures outlined in Table 2 were established for early Phase II testing in a single clinic setting and are intended to change in later phase testing and dissemination to multiple clinic settings. The subsequent feasibility trial (phase II) will measure progress after 3 months among 50 patients randomized to intervention or control. Data will be used to inform future testing (ORBIT phase III-IV) to determine effectiveness using a clinic-randomized controlled trial design with a longer follow-up period.

**Table 2:** Selected outcomes and measures.

**Construct**	**Measurement**	**Number of items (scale)**	**Example item**
**Process measurements**			
Patient's willingness to change behavior	Adapted from the rapid easting assessment for participants (REAPS) survey (72)	2 (Likert scale: very willing to not at all willing)	“How willing are you to make changes in your eating habits in order to be healthier?”
Patient's reason for motivation	The treatment self-regulation scale (75, 76)	11 (Likert scale: very true to not true at all)	“I personally believe that these are important in remaining healthy”
Autonomy-supportive communication	The health care climate questionnaire (short-version) (47)	6 (Likert scale: strongly disagree to strongly agree)	“I feel that the provider has provided me choices and options”
Patient's self-efficacy for healthy food intake and physical activity	The self-efficacy for healthy eating and physical activity measure (SE-HEPA) (77–79)	16 (Likert scale: disagree a lot to agree a lot)	“I can be physically active during my free time on most days even if I have to stay at home”
**Primary Outcomes**			
Improvement in physical activity	Change in minutes of total physical activity: Accelerometry; the international physical activity questionnaire (IPAQ) (80, 81)	4 (numeric)	During the last 7 days, on how many days did you do moderate physical activities like carrying light loads, bicycling at a regular pace, or doubles tennis?
Improvement in healthy food intake	REAPS questions (72) on 5 food intake behaviors: fruit intake, vegetable intake, sugar sweetened beverage consumption, whole grains, and snacking.	5 (yes/no)	Do you eat 2 or more vegetables a day?
Weight stabilization/loss	BMI z score, BMI, height, weight	NA	NA
**RE-AIM implementation outcomes**			
**Reach**			
Percent and representativeness of sample compared to eligible clinic population	EHR data, demographics survey	NA	% black
Patient satisfaction with PREVENT tool	Focus groups	NA	How do you feel about your doctor using PREVENT at your next visit?
**Adoption**			
Percent and representativeness of healthcare team members who use PREVENT	Clinic data	NA	% physicians
Provider satisfaction with PREVENT tool	Provider survey [items adapted from Foraker et al. (65)]	16 (Likert scale: completely disagree to completely agree)	The PREVENT tool is user friendly
**Implementation**			
Acceptability	Acceptability of intervention measure (82)	4 (Likert scale: completely disagree to completely agree)	PREVENT is appealing to me
Appropriateness	Intervention appropriateness measure (82)	4 (Likert scale: completely disagree to completely agree)	PREVENT seems fitting
Feasibility	Feasibility of intervention measure (82)	4 (Likert scale: completely disagree to completely agree)	PREVENT seems implementable
Fidelity	Direct observation of patient–provider interactions, observation checklist	24	Were the slider bars used to demonstrate to the patient how their overall health would change?
**Maintenance**			
Provider's motivation/capacity for sustained use	Adapted from Legare's CPD reaction questionnaire (83–85)	12 (various Likert scales)	I intend to use PREVENT
Organizational capacity for sustained use	Clinical sustainability assessment tool (86, 87)	35 (Likert scale: little or no extent to a very great extent)	The PREVENT tool fits in well with the culture of the team

##### Self-Determination Theory-Based Process Measurements

Three main treatment targets will be quantified using validated survey measures to assess the success of the intervention: patient's willingness to change their physical activity and food intake behaviors, patient's motivation (autonomous vs. controlled regulation), and the provider's use of autonomous supportive patient–provider communication. The patient's willingness to change will be assessed using two questions (one for each behavior) adapted from the Rapid Easting Assessment for Participants (REAPS) survey (77) that ask participants “how willing are you to make changes in your eating habits in order to be healthier?” REAPS has been validated and widely used among adolescents (78, 79). The Treatment Self-Regulation Scale will determine whether the patient's behavior was driven by autonomous or controlled motivation (80, 81). Patients are asked to consider autonomous (e.g., “I personally believe that these are important in remaining healthy”) and controlled (e.g., “other people would be upset with me if I didn't”) reasons and rate them as not at all true to very true (5-point Likert scale). We hypothesize that the PREVENT tool will generate autonomous motivation. The Health Care Climate Questionnaire (short-version) will be used to assess patient's perceptions of the degree to which their healthcare team member used autonomy-supportive vs. controlling language while using the PREVENT tool (50). Provider's use of autonomy-supportive communication is critical for motivating patients to adhere to behavior change recommendations and increases patient well-being. To understand the role of patient's competence or perceived ability, the Self-Efficacy for Healthy Eating and Physical Activity (SE-HEPA) measure developed by Steele et al. (88) will be used to measure the patient's self-efficacy or competence to engage in specific behaviors related to physical activity and healthy eating (89, 90). For example, respondents are asked to indicate on a 5-point scale how much they agree with statements such as “I can eat healthy foods even when unhealthy foods are available” and “I can be physically active during my free time on most days even if I have to stay at home.”

##### Primary Outcomes

Primary outcomes were informed by healthcare team members' definitions of success when treating adolescents with overweight/obesity. The primary outcomes are change in physical activity, healthy food intake, and weight-related outcomes. Improvement in physical activity will be quantified as change in minutes of total physical activity (light, moderate, or vigorous) from baseline to follow-up. Physical activity will be measured objectively using accelerometry (Actigraph GT3X+, Actigraph of Ft. Walton Beach, FL) and subjectively using survey measures adapted from the International Physical Activity Questionnaire (IPAQ) that have been validated for use in adolescents (91, 92). The participant will be instructed to wear the accelerometer on an elasticized belt, on the mid-axillary line for 7 days (including 2 weekend days). A 7-day monitoring protocol provides reliable estimates of children's free-living physical activity behavior (93). Improvement in healthy food intake will be assessed as positive change from baseline to follow-up using questions extracted from the REAPS survey (77). Patient's will be asked how often they are meeting recommendations using a 3-point Likert scale (Never/Rarely to Usually/Often) for the five targeted behaviors: fruit intake, vegetable intake, sugar sweetened beverage consumption, whole grains, and snacking. Change in BMI z-score and percentile from baseline to follow-up will be used to examine weight stabilization and weight loss in participants who received the PREVENT tool vs. a routine care control. Height and weight measured in the clinic will be used to calculate BMI and BMI z-score using the CDC 2000 growth charts. Healthcare team members identified weight stabilization as a realistic and acceptable goal. Research supports that weight loss goals should be realistic and not necessarily attempt to normalize weight. Even modest weight loss has resulted in improvements in CVH (82, 94, 95). Additionally, patients will report whether they are meeting their physical activity and healthy food intake goals at three intervals throughout the intervention using a 5-point Likert Scale (Never to Always).

##### Implementation Outcomes

We seek to speed translation of this tool into practice by collecting mixed methods data regarding the determinants of current and future implementation of the PREVENT tool, guided by the RE-AIM framework (34, 83). Beyond efficacy, the RE-AIM framework focuses measures on the reach of the intervention to a representative proportion of the target population, adoption of the intervention by diverse settings and practitioners, consistent and efficient implementation, and maintenance post intervention (84).

Reach will be examined as percent participation and the representativeness of the study sample compared to the eligible clinic sample. EHR and demographic survey data will be used to determine whether the samples differ demographically (e.g., based on age, gender, race, income, obesity status, geographic location). Additionally, focus group discussions will be conducted to gain in-depth understanding of patients and families perceptions (e.g., satisfaction, usability, acceptability) of the PREVENT tool, which is operationalized as an essential determinant of Reach. Adoption will be assessed as percent and representativeness of healthcare team members who use the tool and their satisfaction with the PREVENT tool. Provider satisfaction with 5 components of the tool (content, accuracy, format, ease of use, and timeliness) will be assessed using a survey specific for HIT interventions (66), adapted for the present study. Implementation will be assessed as healthcare team members' perceived acceptability, appropriateness, and feasibility of the PREVENT tool, as well as fidelity to intended use of the tool. We will use validated measures of acceptability, appropriateness, and feasibility—the Acceptability of Intervention Measure, Intervention Appropriateness Measure, and Feasibility of Intervention Measure (85).

To assess fidelity of implementation, as well as any adaptations (86, 87) made by different settings and staff, direct observation of patient–provider interactions while using the PREVENT tool will generate implementation details. A direct observation checklist will be used by the observer to determine the number of interactions with the PREVENT tool that were delivered as intended (fidelity). These observations will be audio-recorded (in-person or using Zoom technology) and reviewed using qualitative methods to generate further understanding of implementation (e.g., who is delivering the tool, time to administer each step, how the provider delivers the information and adaptations made) and themes in patient–provider communication (e.g., shared decision making, autonomous language). Given the preliminary stage, we will not collect costs, which is a third component of implementation outcomes under RE-AIM. Maintenance will be assessed at the provider (96–98) and clinic levels (99, 100) to understand the potential for sustained use and identify barriers (e.g., organizational, financial, regulatory factors) that may influence sustainability. Early phase implementation data will be used to develop an implementation strategy package that will be tested in later-phase effectiveness-implementation trials (ORBIT Phase III-IV) to enhance the adoption, implementation, local adaptation, and sustainability of PREVENT (101).

#### Refine Prevent Tool (Phase 1b)

Overall, healthcare team members found the tool to be useful, well-organized, and visually appealing (Table 3). The mean user-satisfaction score was 4.6 (*SD* = 0.44) with the average for each user-satisfaction item >4.5 on a 5-point scale (Table 4). This study was not powered to detect significant differences among participants, but there appeared to be no differences in satisfaction scores across setting (primary care vs. specialty clinic) or provider type (e.g., physician, dietician), which suggests that this tool is usable in a variety of care settings and clinical roles. Healthcare team members liked having a display of CVH risk factors accessible at a glance and felt this would facilitate patient discussion. Additional risk factors (e.g., triglycerides, sleep) and functionality (e.g., ability to see previous visit data) were suggested. Automated behavior change recommendations were perceived as patient centered, specific, and achievable; healthcare team members suggested having an option that would allow them to amend (e.g., add open text box to type in goals) the recommendations while interacting with the patient. Healthcare team members noted that having specific examples of foods to suggest to patients would help improve ease of communication and feasibility of goal setting. The ability to tailor activities (e.g., basketball, dance) to meet patient preference was perceived as beneficial for patient motivation. PREVENT's interactive resource map eliminated provider's need to search for or be knowledgeable of resources in a wide range of geographic areas; healthcare team members offered ways to further usability (e.g., color coding resource types).

**Table 3:** Summary of user testing results.

**Themes**	**Key findings**	**Sample quotes**
**Risk profile**		
Purpose	Visually show patients and their families their health status and change that needs to occur to improve overall health	“I would definitely review their BMI…. I would review the risk at the top there based on what information was given.” “So that's reducing that risk at the top…that's helpful so you can show them…you're doing so many minutes of moderate and vigorous activity now, but if you upped it to X number…could show how they could get their health back into the green zone.”
Usefulness	Ability to change risk factors and see change in overall risk Inclusion of patient's current physical activity and food intake behaviors Add clarity to risk factor definitions (e.g., define overall risk, tried smoking, blood pressure)	“These questions [food intake] are pretty good. Those are very specific. ‘Do you eat 2 or more fruits a day?' I think that's pretty good.” “It's very abstract when you do it on a growth chart…the color coding makes more sense, and for us, seeing that 99th makes more sense. Being able to toggle and get it to the yellow, we could say ‘so and so would only need to gain however many inches and stay this weight to get to a healthier BMI.' And that makes sense.”
Usability	Display of risk factors that are otherwise diffuse in the patient's chart Color coding easy to interpret Easy to navigate Nice to be able to turn off risk factors if data is not available Add ability to see previous visit risk factor data	“I like the visual aspect of it…so it's taking all of this information and showing you the overall risk. I think this would be fun…. I think kids and adolescents would like this a lot…it's really helpful for them to see what you're talking about and interact with it…it's easy to click through…we can move through it fairly quickly.” “These are numbers that we don't always have. It looks like we can turn it off.”
**Behavior change recommendations**		
Purpose	Help patient's set individualized, achievable goals	“We could show them the screen and have them pick a couple things they want to work on….I would focus on the greens more than the yellows or the reds.”
Usefulness	Patient-centered content (e.g., food intake goals tailored based on what patient identified as problem areas) Goals are progressive, achievable and easy to understand Helpful for providers who lack knowledge/comfort to develop goals Providers may only have time to discuss the most pressing needs Amend content of recommended food lists	“I like that this populates in based on the questions that they've answered because it's almost like targeting some of their behaviors as opposed to me…telling them things to do…that might get some more buy-in from the families.” “I would love to address every single thing, but I know every single family gets really overwhelmed really fast…so I would probably just pick one of those to do.”
Usability	Organized and user friendly Add ability for provider to modify goals (e.g., add a “write-in” box) or select specific goals to target	“I love that there's examples of foods in here and it's more simplistic in the way it's organized. We have handouts we can add in through the EHR but it's in paragraph form. No kid is going to sit there and read it. So this is actually much more user friendly.”
**Selection of activities**		
Purpose	Help patients select specific activities they enjoy to provide specificity and tailor recommendations to help patients be active	“If they already enjoy something that they don't do very much, that would be a great way to encourage something that they already do. I think that goes back to the sense of self.” “This can help guide us because we're doing this along with the patient….I like this for sure.”
Usefulness	Inclusion of diverse activities (including family-oriented activities) Activities in each activity-level (light, moderate, vigorous) Tool enhances current practice Suggested that activities are culturally appropriate (e.g., feasible in our region)	“Some kids have trouble finding ideas of things they like to do that count as physical activity so it's nice to have things they can choose.” “If the whole family will do these things, that is really, really helpful…go for a walk as a family two times a week…something like that so that. the teen doesn't feel like it's all on them.” “This is so much more detail oriented because we usually say ‘go for a walk. Track your steps on a phone.' This adds more variety of things that they can choose that may be more appealing…this may be more geared to them picking a couple things to attempt even if they haven't done it before.”
Usability	Ability to select activities tailored to each patient Add a “write-in” box to add an activity	“I love that you can tailor the activities and let them pick what they would be willing to do. I think that's awesome.”
**Community resource map**		
Purpose	Add resources near where the patient lives to recommendations and eliminate the need for providers to search for resources	“I think that is one of the coolest things…I never know where anyone lives. They'll tell me their address and I'll be like ‘I don't know where that is.' ‘Where is the WIC office?' ‘I have no idea.' I think that is so great.”
Usefulness	Types of resources included are useful for behavior change Display of resources near the patient's home Inclusion of directions to the resource (by foot, car or public transportation) Eliminates provider need to conduct an online search of resources to offer to patients Add online/internet resources (e.g., dancing games, mindfulness apps)	“If you're trying to prescribe them to get active, knowing that they have a park or a basketball court or something close to them would be helpful.” “I think it's great that the locations are listed like this and they can see how far it is from home.” “We spend a little bit of time in going to websites for families, like printing out a page from a website…that would be nice to have in one place… I love that resources part.”
Usability	Automatic inclusion in the prescription eliminates burden. Make clear what types of resources are included	“…Here you have the categories and maybe next to it, if there could be a category that was color coded or something. I could see that being helpful. It can be hard from the name to tell if it's a farmers market or food pantry or a grocery store.” “It [resources] would be helpful and it would be something that our office staff doesn't have to do because it's automated.”
**Prescription**		
Purpose	Provide patients a firm, detailed plan with community resources to review after the visit	“I think that's really awesome. It definitely gives a firm plan, but it also gives ways for them to get there and resources to help too.”
Usefulness	Design and layout provides clear goals for patients The addition of educational material (e.g., serving size information, tips for being active) Make changes to increase likelihood that patients read this resource (e.g., re-order material, shorten) Amend language to increase patient autonomy	“I think this is great with the green, yellow, red. That's enough of a distinction.” “The question in my mind is how do we reach those patients that don't read a lot of things?…This is longer, if there's one thing they can focus on it's diet, often…so maybe if the food part was up higher that could help.” “The ‘we've suggested some goals for you' doesn't sound like much of a partnership…when it comes from the patient they're going to be more likely to buy in when they think it's their idea. So something…together we try to develop some sort of goals for healthy changes.”
Usability	Electronic delivery to patient (not from provider's email). Be sure information sent is secure. Add way for patient to access resource map from home	“We have a little bit of trouble with emailing because it would come from my email. Not every provider is feeling like they want to send an email to all their patients.”

**Table 4:** Provider satisfaction survey results.

	**Mean (SD)**
The information the tool provides is useful	4.77 (0.44)
The information is presented in a useful form	4.54 (0.52)
The tool was easy to use	4.85 (0.37)
The tool seems possible to use with my patients	4.54 (0.66)
The tool would help me be more effective	4.46 (0.66)
The tool would make the information I want easier to access	4.54 (0.77)
The tool would help meet my needs when providing care for overweight or obese patients	4.54 (0.62)
Total	4.60 (0.44)

*Responses reported on a 5-point Likert scale: strongly disagree (1) to strongly agree (5)*.

The automatic and electronic delivery of a “concrete plan with resources” streamlined care included useful content and was visually appealing. Participants liked the “red, yellow, green” color scheme indicating desired behaviors or outcomes across the tool; they felt that the visual consistency would be easy for patients to understand. Healthcare team members appreciated the ability to print the prescription as part of an after-visit summary and to send information electronically using secure, Health Insurance Portability and Accountability Act of 1996 (HIPAA) compliant communication from an address other than their personal email.

Images and features of the PREVENT tool described above [*Characteristics of the PREVENT Tool (Phase Ia)*] include refinements made based on user-testing results. Changes to the risk portion of the tool included a more descriptive risk title (i.e., changed from “overall risk” to “cardiovascular health score”), addition of the date of health data collection, description of moderate and vigorous activity levels, addition of a “former” smoker option to distinguish from never and current, and description of smoking status categories. We incorporated an option to manually add a physical activity not included in the menu of youth activities portion of the behavior change prescription tab and removed options not relevant to our geographic region (e.g., surfing). Community resources were color coded to distinguish better between types of resources (e.g., grocery stores vs. food pantries). The tool also was programmed to be more compatible with touchscreen-enabled computers, and several buttons/functions (e.g., back arrows, filter for community resource type) were redesigned to be more intuitive to the user.

## Discussion

This first phase of product development identified a clinical problem and developed a solution (the PREVENT tool) that delivers tailored, theory-based behavior change prescriptions and links patients to resources that are desired and accessible to the patient at the point of care. The next phase will test whether the use of informatics and HIT makes this patient- and healthcare team-centered, data-driven approach to behavior change feasible within routine care. The PREVENT tool may be integrated into diverse clinical settings (e.g., pediatric primary care and speciality clinics) to address obesity among children and adolescents while prompting data-informed patient–provider prevention discussions. It is designed to engage and motivate the patient by increasing their autonomy and competence to achieve recommendations for physical activity and healthy food intake (102). The PREVENT tool uses multiple data inputs and outputs to not only inform care practices but systematically generate evidence desired by healthcare teams to demonstrate patient progress.

The primary purpose of this paper was to describe the PREVENT tool and how the ORBIT Model, principles of D4DIS, and behavioral theory were used in its development. While additional testing is necessary to determine the ultimate impact of the PREVENT tool, the use of an intentional, user-centered design process should increase the likelihood of the intended outcomes (e.g., behavior change, weight stabilization/loss) and increase uptake, implementation success, and long-term sustainment and effectiveness (35). Through this process, we designed a novel, pragmatic HIT tool that aids healthcare team members in their existing roles, addresses a clinical need, and overcomes provider-identified pains currently limiting their ability to generate behavior change among adolescents with overweight/obesity.

Healthcare team members were satisfied with the PREVENT tool, felt it was easy to use, and indicated that it would be useful for helping patients with overweight/obesity lead healthier lifestyles. The PREVENT tool is a work in progress that will continue to undergo iterative improvements as we progress through the ORBIT phases of development. We anticipate changes in the provider interface, data input and output modalities, and measurement items. Furthermore, we were unable to incorporate all suggestions from user testing (e.g., inclusion of additional risk factors, EHR integration) into tool updates at this phase due to time and budget constraints. We will continue to track end-user recommendations, as well as gain feedback from patients and families, and incorporate these in future phases of development and testing. Based on the ORBIT model, the PREVENT tool has met the essential milestones for moving to Phase II (preliminary testing): confidence that the treatment package is complete; inclusion of essential components offered in an efficient, feasible, and sustainable way; safe and acceptable to the user of interest (e.g., healthcare teams); and plausible that the treatment will have a clinically significant benefit (35). The deliberate selection of intervention targets has prepared our team to include clinically meaningful targets and implementation outcomes in the next preliminary/pilot testing to prepare for Phase III efficacy testing.

Potential limitations include that the PREVENT tool is limited to children and adolescents who are overweight or obese but may be of interest to those who are sedentary and/or who do not meet dietary recommendations, yet maintain a normal weight. The tool is limited to the English language, yet should be translated to effectively scale to broad populations. Although PREVENT uses informatics and has HIT capabilities, the tool is not yet integrated into the EHR, which may limit workflow compatibility. The PREVENT tool was developed with input from the end user (healthcare teams), yet has not engaged other key stakeholders (e.g., patients and their families, administrators, informaticians, payers). Subsequent testing will elicit patient and families' feedback on the acceptability of the PREVENT tool. We did not include patient/family perspectives to this point in PREVENT development but intend to further refine the tool based on patient and families' desires, as the patient perspective is critical to the success of this intervention. Future work should perform customer discovery with additional stakeholder decision makers who will care about time required and financial and opportunity costs that will ultimately impact dissemination and sustainment. While the tool includes multiple data inputs to tailor recommendations, it does not integrate objective measures of physical activity (e.g., FitBits, accelerometers) that are more reliable indicators of physical activity levels than patient-reported surveys (103). Furthermore, the tool does not collect data or provide feedback on patient use of the community resources. Although the tool assesses an individual patient's motivation/readiness to change physical activity and food intake behaviors, the PREVENT tool does not use this information to automatically prioritize recommendations to behaviors the patients is most ready to tackle.

To our knowledge, the PREVENT tool is one of the only HIT tools that harnesses clinical and community data to help healthcare teams deliver precision prevention (behavior change recommendations tailored to the individual and their community) and reshape the way we care for children and adolescents who are overweight/obese and promote population health (104). Data-centric tools, such as the PREVENT tool, can facilitate individually tailored patient–provider interactions that are necessary to motivate patients to make behavior change, a cornerstone of chronic disease prevention and management (102).

## Data Availability Statement

The datasets presented in this article are not readily available because all original data are qualitative in nature and potentially identifiable. Requests to access the datasets should be directed to Maura M. Kepper, kepperm@wustl.edu.

## Ethics Statement

The studies involving human participants were reviewed and approved by Washington University in St. Louis Institutional Review Board. Written informed consent for participation was not required for this study in accordance with the national legislation and the institutional requirements.

## Author Contributions

MK and CW-B wrote, edited the manuscript, collected, and analyzed data. RB, BK, EM, JG, LF, RG, ML, and RF contributed to the editing of the manuscript. MK, CW-B, ML, and RF contributed to the development of the tool. All authors contributed to the article and approved the submitted version.

## Conflict of Interest

The authors declare that the research was conducted in the absence of any commercial or financial relationships that could be construed as a potential conflict of interest.
